# The influence of background diabetic retinopathy in the second eye on rates of progression of diabetic retinopathy between 2005 and 2010

**DOI:** 10.1111/aos.12074

**Published:** 2013-04-01

**Authors:** Peter H Scanlon, Irene M Stratton, Mark Histed, Steve J Chave, Stephen J Aldington

**Affiliations:** 1 Gloucestershire Diabetic Retinopathy Research Group, Cheltenham General HospitalCheltenham, UK; 2 The NHS Diabetic Eye Screening ProgrammeGloucester, UK

**Keywords:** diabetic retinopathy, progression, risk factors, screening

## Abstract

Purpose: The Gloucestershire Diabetic Eye Screening Programme offers annual digital photographic screening for diabetic retinopathy to a countywide population of people with diabetes. This study was designed to investigate progression of diabetic retinopathy in this programme of the English NHS Diabetic Eye Screening Programme.

Methods: Mydriatic digital retinal photographs of people with diabetes screened on at least 2 occasions between 2005 and 2010 were graded and included in this study if the classification at first screening was no DR (R0), background DR in one (R1a) or both eyes (R1b). Times to detection of referable diabetic retinopathy (RDR) comprising maculopathy (M1), preproliferative (R2) or proliferative retinopathy (R3) were analysed using survival models.

Results: Data were available on 19 044 patients, 56% men, age at screening 66 (57–74) years (median, 25th, 75th centile). A total of 8.3% of those with R1a and 28.2% of those with R1b progressed to any RDR, hazard ratios 2.9 [2.5–3.3] and 11.3 [10.0–12.8]. Similarly 7.1% and 0.11% of those with R1a progressed to M1 and R3, hazard ratios 2.7 [2.3–3.2] and 1.6 [0.5–5.0], compared to 21.8% and 1.07% of those with R1b, hazard ratio 9.1 [7.8–10.4] and 15.0 [7.1–31.5].

Conclusions: The risk of progression is significantly higher for those with background DR in both eyes than those with background retinopathy in only one or in neither eye.

## Introduction

The current recommendation of the NHS Diabetic Eye Screening Programme is for all people with diabetes aged 12 years or over to be screened with digital retinal photographs each year until they have evidence of sight threatening diabetic retinopathy, which is defined by the referable criteria used by the English NHS Diabetic Eye Screening Programme. The referable criteria are the presence of features of maculopathy (M1), preproliferative (R2) or proliferative retinopathy (R3) or a combination of these features, as defined in the methods section. Presence of any evidence of referable diabetic retinopathy (RDR) results in referral to the Hospital Eye Service.

Patients with only mild background retinopathy continue to be managed in the same way as those with no evidence of diabetic retinopathy and receive annual screening.

Here, we compare the risk of progression to RDR in those with mild background retinopathy in one eye, in both eyes and those with no DR. We also specifically compared the risk of progression to maculopathy (M1) and proliferative retinopathy (R3) and a combination of preproliferative (R2) and proliferative retinopathy (R3) in the same groups.

## Materials and Methods

The Gloucestershire Diabetic Eye Screening Service delivers retinal screening through a mobile screening programme in 85 primary care practices. Two field 45° digital retinal images (macular and disc centred) are graded in a central office by an experienced quality assured team.

The criteria used for grading in the Gloucestershire Diabetic Eye Screening Programme and the relationship to the Early Treatment Diabetic Retinopathy Study (ETDRS) severity scale (ETDRS 1991) are described below:

R0 level identifies no detected diabetic retinopathy (equivalent to ETDRS level 10).

R1 level (mild NPDR or background DR) identifies a minimum of at least the presence of one microaneurysm and/or retinal haemorrhage, equivalent to ETDRS levels 20–35. R1a defined the presence of these features in only one eye and R1b the presence in both eyes.

R2 level (moderate to severe NPDR or preproliferative DR) identifies the presence of multiple deep, round or blot haemorrhages and/or definite intraretinal microvascular abnormality (IRMA) and/or venous beading and/or reduplication, equivalent to levels 43–53 on the ETDRS scale.

R3 level (proliferative DR) indicates the presence of proliferative diabetic retinopathy (including fibrous proliferation), equivalent to a minimum of ETDRS level 61.

M1 (maculopathy) identifies the presence of 2-dimensional photographic markers of diabetic maculopathy, specifically exudate within 1 disc diameter (DD) of the centre of the fovea, circinate or group of exudates within the macula or any microaneurysm or haemorrhage within 1DD of the centre of the fovea but only if associated with a best VA of worse than 0.3 logMAR (equivalent to Snellen 6/12).

M0 describes the absence of any M1 features.

Patients were categorized into groups on the basis of the presence of retinopathy in neither, one or both eyes at each of the two baseline screening episodes. Patients with unassessable images, with any evidence of previous laser treatment or with features of sight-threatening diabetic retinopathy (STDR) at either baseline screening event were excluded. Sight-threatening diabetic retinopathy was defined by the presence of any R2 (moderate to severe NPDR), R3 (proliferative DR) or M1 (maculopathy) in either eye.

We reviewed all patients with assessable images of both eyes from at least two screening episodes between January 2005 and December 2010. We included those who at the first (index) screening had no retinopathy or only mild background retinopathy in one or both eyes. They were followed up until referable retinopathy was detected or until no more screening episodes were available.

Life table survival plots were derived using Kaplan–Meier estimates. Hazard ratios were calculated using Cox Proportional Hazards models. Because the data were interval censored (screening being carried out at approximately yearly intervals), the models were fitted using semi-closed intervals in PROC PHREG in SAS. All data were analysed using SAS 9.1 (SAS Institute Inc., Cary, NC, USA).

## Results

Data were available on 19 044 patients (see Table 1) and 10 705 (56.2%) were men. The median age at first screening (years) was 66 (57–74) The median LogMAR visual acuity in the better eye was 0.06 (0.00–0.20) The median time from first screening to rescreening (months) was 13.5 (12–15) The median follow-up (months) was 41 (24–53).

**Table 1:** The influence of baseline screening result on subsequent screening referral.

*N* = 19 044					Median (25th, 75th centile)				
Male					10705 (56.2%)				
Age at index screening (years)					66 (57–74)				
LogMAR visual acuity in better eye					0.06 (0.00–0.20)				
Time from index screening to rescreening (months)					13.5 (12–15)				
Follow-up (months)					41 (24–53)				
		Number progressing to referable DR (RDR), maculopathy (M1), preproliferative (R2) or proliferative DR (R3), or to proliferative DR (R3 alone).				Hazard ratios for the development of each referable category			
	No in group	RDR*	M1	R2 or R3	R3	RDR	M1	R2 or R3	R3
No DR	12491	399 (3.19%)	361 (2.89%)	74 (0.59%)	9 (0.07%)	1	1	1	1
R1 in 1 eye (R1a)	3744	312 (8.33%)	267 (7.13%)	96 (2.56%)	4 (0.11%)	2.9 (2.5–3.3)	2.6 (2.3–3.1)	4.6 (3.4–6.2)	1.6 (0.5–5.0)
R1 in both eyes (R1b)	2809	792 (28.2%)	611 (21.8%)	446 (15.8%)	30 (1.07%)	11.3 (10.0–12.8)	9.1 (7.9–10.4)	30.7 (24.0–39.3)	14.9 (7.1–31.5)
				No DR		R1 in 1 eye (R1a)		R1 in both eyes (R1b)	
No in group				12 491		3744		2809	
Male (%)				6928 (55.5%)		2172 (58.0%)		1606 (57.2%)	
Age at index screening (years)				66 (57–74)		67 (58–75)		66 (56–75)	
LogMAR visual acuity in better eye				0.06 (0.0–0.2)		0.06 (0–0.2)		0.06 (0–0.2)	
Time from index screening to rescreening (months)				13.5 (12.0–15.2)		13.4 (12.0–15.2)		13.7 (12.1–15.4)	
Follow-up (months)				46 (26–53)		41 (24–53)		39 (24–52)	

* As individual patients could develop a combination of M1, R2 or R3, the sum of these columns will not be the same as the total RDR.

A total of 8.3% of those with R1a and 28.2% of those with R1b progressed to RDR, hazard ratios 2.9 (2.5–3.3) and 11.3 (10.0–12.8). 7.1% of those with R1a progressed to M1, hazard ratios 2.7 (2.3–3.1), compared to 21.8% with R1b, hazard ratio 9.1 (7.9–10.4). 2.56% of those with R1a progressed to R2 or R3, hazard ratio 4.6 (3.4–6.2), compared to 15.8% of those with R1b, hazard ratio 30.7 (24.0–39.3). 0.11% of those with R1a progressed to R3, 1.6 (0.5–5.0), compared to 1.07% of those with R1b, hazard ratio 15.0 (7.1–31.5).

**Figure 1:**
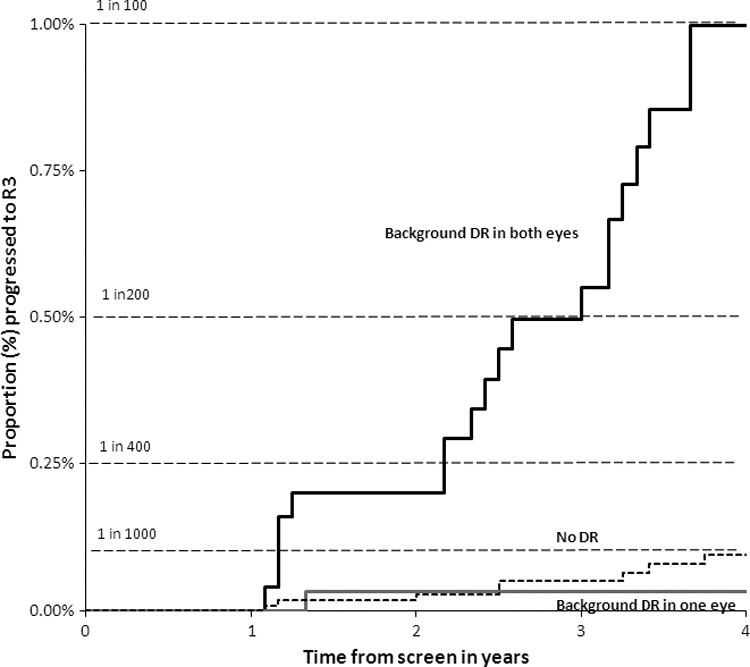
Time to proliferative DR (R3) from screen with no referable DR.

**Figure 2:**
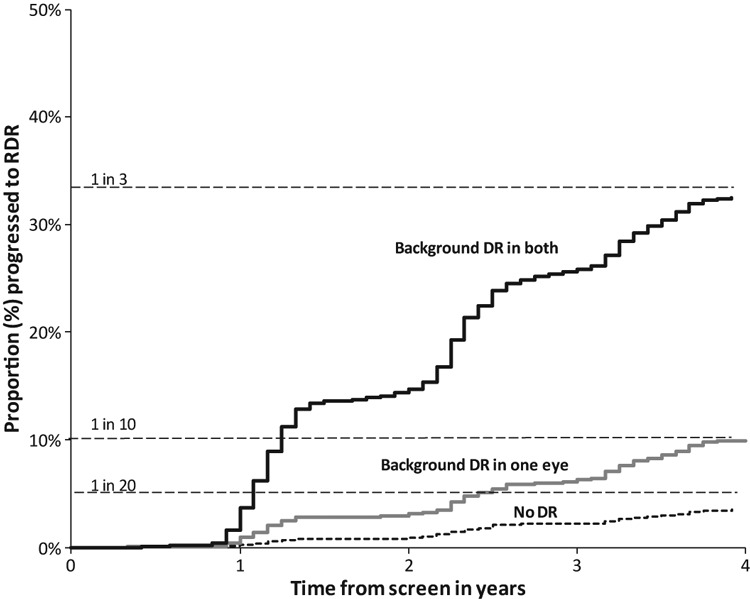
Time to referable DR (RDR) from screen with no referable DR. [Correction added on 11 April after online publication to reflect the correct figure legend. It was previously reported that the figure describes the time to proliferative DR (R3) from screen with no referable DR.]

**Figure 3:**
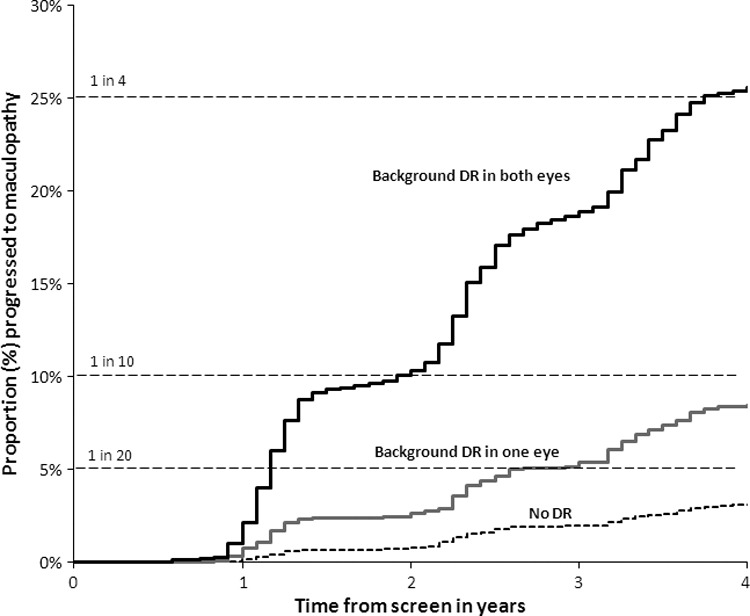
Time to maculopathy (M1) from screen with no referable DR.

**Figure 4:**
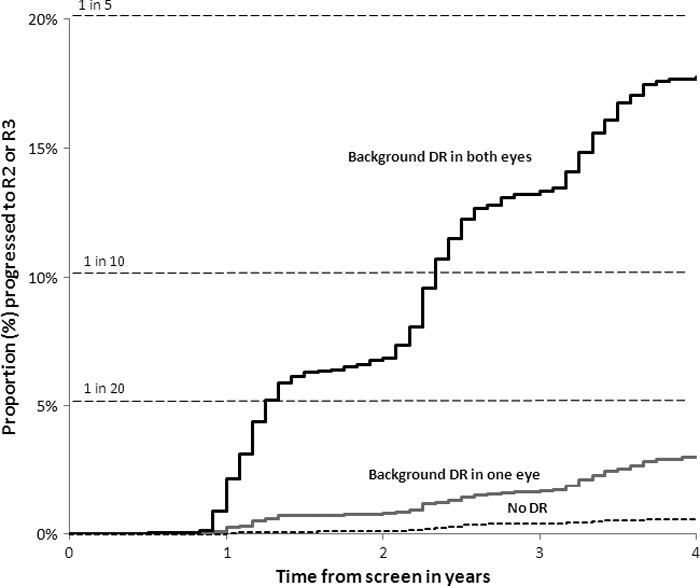
Time to R2 or R3 (pre-proliferative or proliferative DR) from screen with no referable DR.

The rates of progression are shown in Figs 1–4, which demonstrate that the risk of progression is significantly higher (p < 0.001) for those with background DR in both eyes than those with background retinopathy in one only or in neither eye.

An analysis was carried out by age group (patients partitioned into 10-year age groups). Within each age group, the risks observed overall were seen, with the HR for progression in group 1b (R1 in both eyes) being highly significant in all groups (p < 0.001) and because of the smaller numbers significant for group 1a (R1 in 1 eye) p < 0.01 in all age groups.

## Discussion

In 1986, Kohner (Kohner & Sleightholm 1986) demonstrated that there is a relationship between microaneurysm count and severity of diabetic retinopathy. The United Kingdom Prospective Diabetes Study (UKPDS, Aldington et al. 1995) and the Wisconsin Epidemiological Study (Klein et al. 1995) both demonstrated the relationship between retinal microaneurysm count and progression of diabetic retinopathy.

A later report (Kohner et al. 1999) from UKPDS reported that the presence of microaneurysms alone, and also the number of microaneurysms had a high predictive value for worsening retinopathy at 3, 6, 9 and 12 years after entry into the study.

In 2001, Kohner (Kohner et al. 2001) reported the relationship in UKPDS between the severity of retinopathy and progression to photocoagulation in patients with Type 2 diabetes mellitus in the UKPDS.

Nunes (Nunes et al. 2009) reported a relationship between high microaneurysm formation rate on colour fundus photographs and DR progression to clinically significant macular oedema in type 2 diabetic patients with non-proliferative DR.

More recent studies have shown that the rate of progression of diabetic retinopathy has slowed in more recent time periods. This may reflect earlier diagnosis of Type 2 diabetes, improved control of hyperglycaemia and treatment of hypertension in Type 1 and Type 2 diabetes. Differences in baseline characteristics, age (UKPDS recruitment stopped at 65), ethnicity and particularly in the prevalence and severity of retinopathy, could also have contributed to these differences.

The current study included 19 044 people with diabetes and was designed to test the influence of baseline DR and to report demographic characteristics in relation to progression to referable or proliferative diabetic retinopathy from at least two screening episodes between January 2005 and December 2010. It is likely that confounding factors are present, those with background DR are likely to have higher levels of HbA1c and to have had diabetes for longer. However, in this study, we have not included these data as they are not routinely available in screening programmes, certainly not in the English programme. Because this screening programme continues to screen patients once they have been referred to an ophthalmologist the problem of competing risks is minimized. Progression from R2 ro R3 is unlikely to be affected by referral as it is not routine to treat R2 and advice to maintain good glucose control is unlikely to have a large effect in the next 12 or 24 months.

This study has shown that the risk of progression to referable DR is significantly higher for those with background DR in both eyes than those with background DR in 1 eye only or with no DR. This effect was seen in all age groups. Reports from the UKPDS (Kohner et al. 1999, 2001) demonstrated that the number of microaneurysms had a high predictive value for worsening retinopathy and that there was a greater likelihood of having laser treatment over the following 9 years if there were microaneurysms in both eyes at baseline as opposed to one eye alone. However, involvement of a second eye with background DR in this study does appear to have a greater effect than we had anticipated.

The cumulative 4-year risk of progression to proliferative DR is less than one in 1000 for those with no DR or in 1 eye only but was found in one in 200 of those with background DR in both eyes. Similarly, the cumulative 4-year risk of development of referable DR was 1 in 3 for those with background DR in both eyes but less than 1 in 10 and 1 in 20 in those with only one or neither eye affected.

High-quality photographic screening and grading and diligent recording of early lesions of retinopathy are essential to enable classification of risk. This is only possible when the photography and grading is carried out in a quality assured environment by well trained and motivated staff.

The results of this study may help to design and power clinical trials testing interventions such as new drug treatments for diabetic retinopathy. Many clinical trials require a two-step progression per eye or three-step progression per patient on the ETDRS severity scale (Chew 2001; ETDRS 1991). The majority of individual eyes that have progressed from R1 mild non-proliferative diabetic retinopathy to R2 moderate to severe non-proliferative diabetic retinopathy in this study will have progressed by the equivalent of two steps and counting both eyes, by the equivalent of three steps on the ETDRS severity scale. If patients were selected who had bilateral background DR or mild NPDR, we have demonstrated an overall progression to R2 or R3 of 15.9% after 4 years. A trial of an intervention for diabetic retinopathy would benefit from entry criteria of background or mild non-proliferative diabetic retinopathy in two eyes. The intervention is clearly more likely to see an effect on two step progression than with entry criteria of background or mild non-proliferative diabetic retinopathy in only one eye. This would mean that a reduced sample size of patients would be needed within the trial.

The latest figures from the Department of Health (DH 2011) in England show that in the last 12 months, 2.5 million people were identified with diabetes, 2.3 million people were offered screening and 1.8 million people were actually screened. There is currently an epidemic of diabetes in the world, and this is putting a strain on the national screening services that are currently operating, and there is a strong argument for optimizing screening intervals based on individual risk. This risk may be based on a previous screening result (Agardh & Tababat-Khani 2011; Chalk et al. 2012; Olafsdottir & Stefansson 2007; Younis et al. 2003a, b) or on the basis of previously defined individual risk factors (Mehlsen et al. 2011, 2012). The results of this study provide background data on current levels of risk based on one screening result as compared to a recent study (Stratton et al. 2012) in our unit based on the results of two consecutive screening results. England, Scotland Wales and Northern Ireland are currently looking at the latest evidence of risk based on what data is readily available within the health information systems of the four countries to develop a unified approach to this optimization of screening intervals.
